# Continuously wavelength-tunable passband-flattened fiber comb filter based on polarization-diversified loop structure

**DOI:** 10.1038/s41598-017-06952-z

**Published:** 2017-08-16

**Authors:** Jaehoon Jung, Yong Wook Lee

**Affiliations:** 1 Department of Electronics and Electrical Engineering, Dankook University, Gyeonggi-do, Yongin 16890 South Korea; 2 Interdisciplinary Program of Biomedical Mechanical & Electrical Engineering and School of Electrical Engineering, Pukyong National University, Yongso-ro 45, Nam-Gu, Busan 48513 South Korea

## Abstract

Continuous wavelength tuning of optical comb filters, which is an essential functionality for flexible signal processing in reconfigurable optical systems, has been challenging in high order filter structures with two birefringent elements (BEs) or more due to cumbersomeness in finding a combination of waveplates and BEs and complexity in determining their individual azimuthal orientations. Here, we propose a continuously tunable polarization-independent passband-flattened fiber comb filter with two BEs using a polarization-diversified loop structure for the first time. The proposed filter consists of a polarization beam splitter and two groups of a half-wave plate, quarter-wave plate, and polarization-maintaining fiber (PMF). The azimuthal orientation of PMF in the second group is fixed as 22.5°. Orientation angle sets of the four waveplates, which can induce an arbitrary phase shift from 0 to 2*π* in the passband-flattened transmittance function, are found from the filter transmittance derived using Jones matrix formulation. From theoretical spectral analysis, it is confirmed that passband-flattened comb spectra can be continuously tuned. Theoretical prediction is verified by experimental demonstration. Moreover, the wavelength-dependent evolution of the output state of polarization (SOP) of each PMF is investigated on the Poincare sphere, and the relationship between wavelength tuning and SOP evolution is also discussed.

## Introduction

Due to simple design, ease of use, and good fiber compatibility, fiber comb filters have been considered as useful wavelength-selective elements that can be employed to route and process optical signals or block unwanted signals causing crosstalks in dense wavelength-division-multiplexed (DWDM) optical networks. They can also be applied to multiwavelength fiber lasers or waveband sources^1–4^, microwave photonic filters^5, 6^, optical pulse train generation^7^, and so forth. Continuous wavelength tunability of a comb filter is highly beneficial to its efficiency when the filter is involved to select the desired wavelength component or prevent interference between wavelength components in wavelength-routing devices or multiwavelength sources. Numerous efforts have been made to provide a comb filter with wavelength tunability by incorporating a Sagnac birefringence loop (SBL)^8, 9^, a double-loop Mach-Zehnder interferometer (MZI)^10–12^, a Lyot-type birefringence filter^1, 13, 14^, fiber gratings^15, 16^, and a polarization-diversified loop structure (PDLS)^17–20^. Among various approaches, comb filters based on the PDLS^21, 22^, which is constructed using a polarization beam splitter (PBS), have great advantages in terms of effective and flexible switching or tuning capability of their passbands^17–20, 23^. That is partly because an SBL-based comb filter, which uses a directional coupler, suffers from degradation in extinction ratio (ER) during its passband tuning^8, 9, 24^. In particular, PDLS-based comb filters are more resistant to external perturbations such as heat and vibration in comparison with MZI-based ones^10–12^ and also polarization independent unlike Lyot-type birefringence filters^1, 13, 14^. The resonances of fiber gratings can be frequency-shifted using physical stimuli such as tension, pressure, and heat. However, stress accumulated in the fiber gratings during the wavelength tuning operation may do serious harm to their durability and reliability, accompanied with the degradation of their insertion loss (IL), channel isolation, and passband bandwidth.

In the case of a PDLS-based zeroth-order comb filter that employs one polarization-maintaining fiber (PMF) segment as a birefringent element (BE), the continuous spectral tuning has already been implemented using some waveplate combinations (WPCs) such as a group of a half-wave plate (HWP) and a quarter-wave plate (QWP), a group of a QWP and an HWP, and two QWPs^17, 20^. The adjustment of the azimuth angles of waveplates in these WPCs can create a continuous absolute phase change of 0 to 2*π* to the filter transmittance. In particular, a PDLS-based first-order comb filter, formed by connecting two segments of PMF with some special combination rules in the relative orientation angle and length difference, can provide unique transmittance features such as flat-top or narrow passbands. PDLS-based first-order Solc- and Lyot-type comb filters were proposed by concatenating two PMF segments of equal length with an angle offset of 45° between the principal axes of the two segments^25^ and by splicing two PMF segments, one of which was two times longer than the other, with a 60° offset between their principal axes^26^, respectively. Moreover, frequency interleaving operation was realized in both flat-top and narrow band modes of the PDLS-based first-order comb filter by adjusting the relative angular difference between the principal axes of two PMF segments with an HWP sandwiched between them^27^. However, continuous frequency tuning of the PDLS-based passband-flattened comb filter, which requires synchronous modulation of an additional phase difference of 0 to 2*π* between two orthogonal modes of each PMF for two PMF segments, has not been reported until now. As an intuitive but costly way, some differential group delay modulators, or birefringence modulators, were utilized to realize a polarization-dependent Lyot-type comb filter capable of switching or tuning of the free spectral range (FSR), i.e., the channel spacing, and the channel bandwidth in first- and second-order transmittance functions^28^. However, there was no polarization-independent passband-flattened fiber comb filter, which could offer continuous wavelength tunability using BEs with fixed birefringence, due to difficulty in finding a combination of waveplates and BEs (i.e., PMF segments) and complexity in determining their individual azimuthal orientations.

Here, we propose and experimentally demonstrate a continuously tunable polarization-independent passband-flattened fiber comb filter based on the PDLS. The proposed filter is composed of a four-port PBS for implementing the PDLS and two BE groups of an HWP, a QWP, and PMF. The lengths of the two PMF segments are equal. One end of PMF in the second BE group is butt-coupled to one port of the PBS so that its slow axis should be oriented at 22.5° with respect to the horizontal axis of the PBS, and its other end is connected to the QWP in the second BE group. To the best of our knowledge, this is the first demonstration of a polarization-independent passband-flattened fiber comb filter, whose spectral tuning can be continuously performed using two PMF segments and some waveplates instead of sumptuous birefringence modulators^18, 28^. First, orientation angle sets of the four waveplates, which can induce an arbitrary phase shift from 0 (0°) to 2*π* (360°) in the passband-flattened transmittance function, are found from the filter transmittance derived using Jones matrix formulation. From theoretical transmission spectra obtained at eight selected orientation angle sets of the four waveplates, which cause phase shifts increasing linearly from 0 (0°) to 7*π*/4 (315°) by a step of *π*/4 (45°), it is confirmed that the passband-flattened comb spectrum can be continuously tuned by properly controlling the waveplate orientation angles. Then, this theoretical prediction is verified by experimental demonstration. It is experimentally proved that the orientation angles of the four waveplates, which induce an arbitrary phase shift from 0 to 2*π* in the transmittance function, can always be found, that is, the fabricated filter can be continuously tuned by appropriately controlling the waveplate orientation angles. Moreover, the wavelength-dependent evolution of the output state of polarization (SOP) of each PMF segment is explored for the eight selected waveplate angle sets in terms of the Poincare sphere. The relationship between continuous wavelength tuning and SOP evolution is also discussed.

## Principles of operation

**Figure 1:**
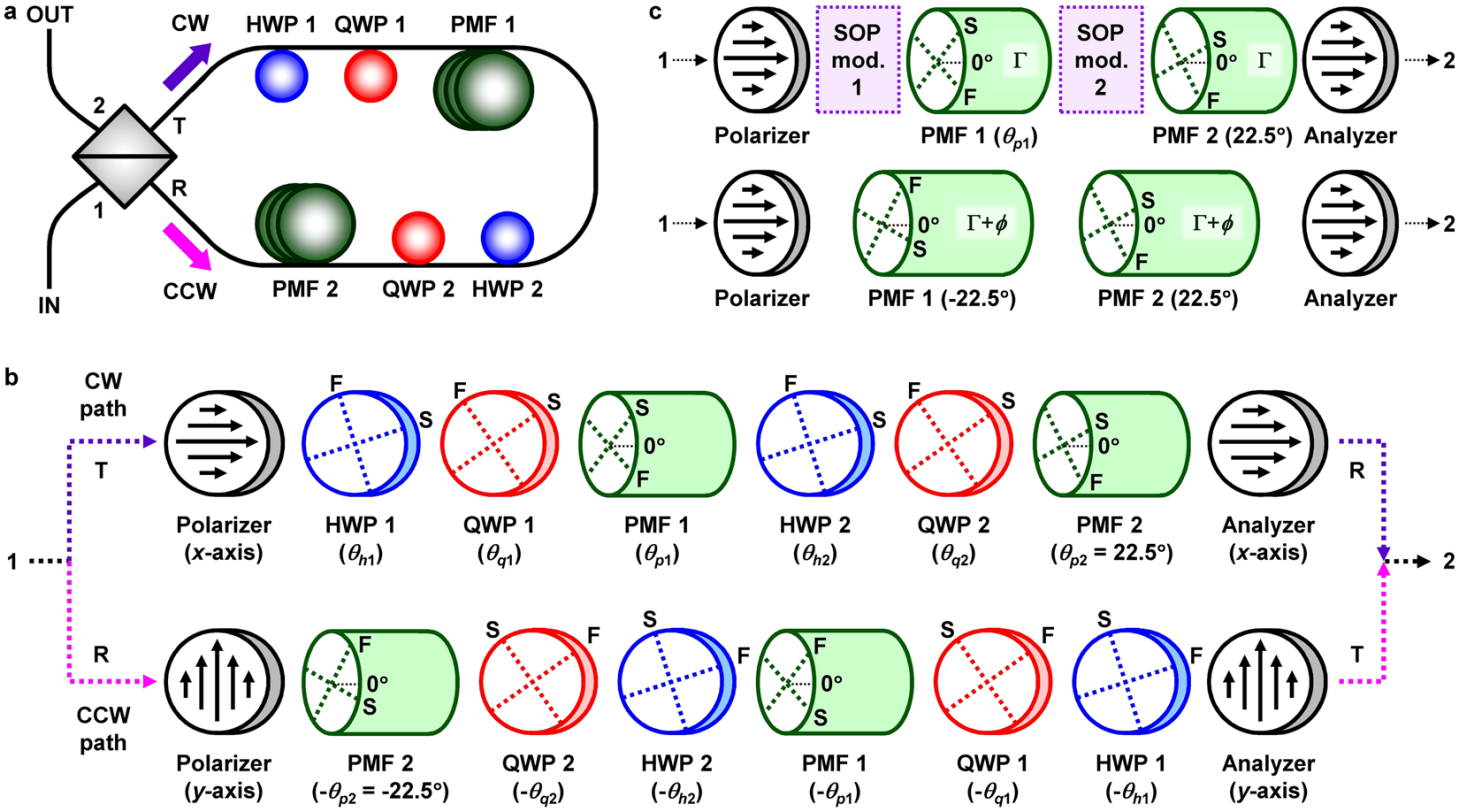
Schematic illustrations of (**a**) proposed filter, (**b**) optical propagation paths of input beam introduced into filter, and (**c**) equivalent arrangement of birefringent elements for continuous tuning of passband-flattened transmission spectrum. The orientation angle of each component is indicated in parentheses. The equivalent arrangement, i.e., the modification of effective phase difference and orientation angles, can be embodied through the modulation of the SOP_in_ of each PMF.

Figure 1(a) shows a schematic diagram of the proposed filter comprised of a four-port PBS and two BE groups of an HWP, a QWP, and PMF, denoted by HWP 1, QWP 1, and PMF 1 in the first BE group and HWP 2, QWP 2, and PMF 2 in the second BE group, respectively. The lengths of PMF 1 and PMF 2 are equal. One end of PMF 2 is butt-coupled to R port of the PBS so that the slow axis of PMF 2 should be oriented at 22.5° for the horizontal axis of the PBS, and the other end is connected to QWP 2. Input light introduced into port 1 of the PBS is decomposed into linear horizontal polarization (LHP) and linear vertical polarization (LVP) components, which propagate through the polarization-diversified loop of the filter in clockwise (CW) and counterclockwise (CCW) directions, respectively. Basically, in polarization-interference-based comb filters, an interference fringe is created by the phase difference Γ (=2*πBL*/*λ*) between two orthogonal polarization modes of PMF, where *B*, *L*, and *λ* are the PMF birefringence, PMF length, and wavelength in vacuum, respectively. This fringe spectrum can be shifted by giving an additional phase difference of 0 to 2*π* to this phase difference, that is, varying the effective birefringence of PMF, through the use of some waveplates^23^.

Figure 1(b) shows the optical propagation path of an input beam passing through the filter. Input light entering port 1 of the PBS is separated into two linearly polarized beams with SOPs of LHP and LVP, which emerge from ports T and R of the PBS, respectively. For convenience, let us assume the horizontal and vertical axes of the PBS as *x*- and *y*-axes in Fig. 1(b), respectively. Along the CW path, the LHP component sequentially passes through a horizontal polarizer (*x* axis), HWP 1 (with its slow axis oriented at *θ*_*h*1_ for the *x* axis), QWP 1 (*θ*_*q*1_ oriented), PMF 1 (*θ*_*p*1_ oriented), HWP 2 (*θ*_*h*2_ oriented), QWP 2 (*θ*_*q*2_ oriented), PMF 2 (*θ*_*p*2_ = 22.5° oriented), and a horizontal analyzer (*x* axis). Similarly, along the CCW path, the LVP component propagates through a vertical polarizer (*y* axis), PMF 2 (−22.5° oriented), QWP 2 (−*θ*_*q*2_ oriented), HWP 2 (−*θ*_*h*2_ oriented), PMF 1 (−*θ*_*p*1_ oriented), QWP 1 (−*θ*_*q*1_ oriented), HWP 1 (−*θ*_*h*1_ oriented), and a vertical analyzer (*y* axis) in turn. Here, F and S indicate the fast and slow axes, respectively, of BEs such as waveplates and PMF. In both CW and CCW paths, an interference spectrum with an equal FSR is generated due to polarization interference, but the IL of each interference spectrum depends on input polarization^23^. Because the two output interference spectra generated in both paths are orthogonally polarized (i.e., LHP and LVP) with each other, the output spectrum of the filter can be obtained by the optical power superposition of the two interference spectra. As arbitrarily polarized light is given by the linear superposition of LHP and LVP components, therefore, the filter output spectrum is independent of input polarization^23^.

In a polarization interference spectrum generated in one PMF segment sandwiched by two polarizers, a transmittance function is given by a sinusoidal function of Γ, which is defined as the zeroth-order transmittance. In case the number of PMF segments employed for polarization interference is *N* (≥2), a transmittance function may contain terms like cos^*N*^Γ, cos^*N*−1^Γ, ···, cosΓ, which is denoted by the (*N*−1)th-order transmittance^29^. The first-order comb filter utilizes two PMF segments, and its first-order transmittance necessarily includes a term “cos^2^Γ”. The zeroth-order transmittance can be spectrally shifted by varying the effective birefringence of PMF, that is, adding an extra phase difference *ϕ* to the original phase difference Γ^20^. Similarly, in the case of the first-order transmittance, an additional phase difference *ϕ* should be simultaneously introduced into Γ of each PMF for its spectral tuning. For example, in order to tune a passband-flattened transmission spectrum, a representative spectrum of the first-order comb filter, the effective phase difference of each PMF segment should be Γ + *ϕ* with the effective orientation angles of PMF 1 and PMF 2 to be −22.5° and 22.5°, respectively. This modification of the effective phase difference and orientation angles can be embodied by modulating the input SOP (SOP_in_) of each PMF, as shown in Fig. 1(c). In our study, this SOP_in_ modulation is carried out using a WPC of an HWP and a QWP per each PMF. While this additional phase difference *ϕ* changes from 0 to 2*π*, the flat-top comb spectrum moves in the wavelength domain by one FSR, that is, is fully tuned over a channel spacing.

On the basis of Jones matrix formulation^30^, the theoretical transmittance of the proposed filter is derived using the transfer matrix *T*. In this derivation, the ILs of the waveplates, PMF segments, and PBS are not considered, and the waveplates are also assumed to be independent of wavelength. Detailed expression on *T* and the transfer matrices of PMF and waveplates can be found in part 1 of Supplementary Information online. The filter transmittance *t* shown in (1) can be obtained from *T* for arbitrary input polarization^23^.

1 $$\begin{array}{rcl}t & = & \frac{1}{4}[{A}_{1}^{2}+{A}_{2}^{2}+2({A}_{1}{B}_{1}+{A}_{2}{B}_{2})\cos \,{\rm{\Gamma }}+2(-{A}_{1}{C}_{1}+{A}_{2}{C}_{2})\sin \,{\rm{\Gamma }}\\  &  & \,+({B}_{1}^{2}+{B}_{2}^{2}){\cos }^{2}{\rm{\Gamma }}+2(-{B}_{1}{C}_{1}+{B}_{2}{C}_{2})\sin \,{\rm{\Gamma }}\,\cos \,{\rm{\Gamma }}+({C}_{1}^{2}+{C}_{2}^{2}){\sin }^{2}{\rm{\Gamma }}]\end{array}$$

where *A*_1_ = sin(2*θ*_*p*1_ − 2*θ*_*h*2_−*θ*_*q*1_ + *θ*_*q*2_)cos(2*θ*_*h*1_−*θ*_*q*1_ + *θ*_*q*2_ + 45°) + sin(2*θ*_*h*1_−*θ*_*q*1_−*θ*_*q*2_)sin(2*θ*_*h*2_−*θ*_*q*1_−*θ*_*q*2_), *B*_1_ = sin(2*θ*_*h*1_ − *θ*_*q*1_ − *θ*_*q*2_)sin(2*θ*_*h*2_ − *θ*_*q*1_ − *θ*_*q*2_) − sin(2*θ*_*p*1_−2*θ*_*h*2_−*θ*_*q*1_ + *θ*_*q*2_)cos(2*θ*_*h*1_−*θ*_*q*1_ + *θ*_*q*2_ + 45°), *C*_1_ = cos(2*θ*_*p*1_ − 2*θ*_*h*2_ − *θ*_*q*1_ + *θ*_*q*2_)cos(2*θ*_*h*1_ − *θ*_*q*1_ − *θ*_*q*2_) + cos(2*θ*_*h*2_ − *θ*_*q*1_ − *θ*_*q*2_)sin(2*θ*_*h*1_ − *θ*_*q*1_ + *θ*_*q*2_ + 45°), *A*_2_ = cos(2*θ*_*p*1_ − 2*θ*_*h*2_ − *θ*_*q*1_ + *θ*_*q*2_)sin(2*θ*_*h*1_ − *θ*_*q*1_ − *θ*_*q*2_ − 45°) + cos(2*θ*_*h*1_ − *θ*_*q*1_ + *θ*_*q*2_)cos(2*θ*_*h*2_ − *θ*_*q*1_ − *θ*_*q*2_), *B*_2_ = cos(2*θ*_*h*1_ − *θ*_*q*1_ + *θ*_*q*2_)cos(2*θ*_*h*2_ − *θ*_*q*1_ − *θ*_*q*2_) − cos(2*θ*_*p*1_ − 2*θ*_*h*2_ − *θ*_*q*1_ + *θ*_*q*2_)sin(2*θ*_*h*1_ − *θ*_*q*1_ − *θ*_*q*2_ − 45°), and *C*_2_ = sin(2*θ*_*p*1_ − 2*θ*_*h*2_ − *θ*_*q*1_ + *θ*_*q*2_)sin(2*θ*_*h*1_ − *θ*_*q*1_ + *θ*_*q*2_) + sin(2*θ*_*h*2_ − *θ*_*q*1_ − *θ*_*q*2_)cos(2*θ*_*h*1_ − *θ*_*q*1_ − *θ*_*q*2_ − 45°). *θ*_*h*1_, *θ*_*q*1_, *θ*_*p*1_, *θ*_*h*2_, *θ*_*q*2_, and 22.5° are the slow-axis orientation angles of HWP 1, QWP 1, PMF 1, HWP 2, QWP 2, and PMF 2 with respect to the *x* axis, respectively. Here, *θ*_*h*1_ and *θ*_*h*2_ have an angle symmetry of 180°. Among diverse first-order spectra obtainable using this transmittance, a passband-flattened transmittance function *t*_*f*_, which corresponds to the complemented transmittance of a fan Solc filter with two birefringent plates (BPs)^31^, is given by (2). A conventional fan Solc filter used as a narrowband filter is composed of two parallel polarizers between which *M* BPs with equal phase difference Γ are inserted, and each BP is positioned with its slow axis oriented at an increasing sequence *θ*, 3*θ*, 5*θ*, …, and (2 *M−*1)*θ* with respect to the polarizing axis of the input polarizer, where *θ* = *π*/4 *M*. The unity transmission occurs only at the wavelength where the BPs have an even number of half-waves of retardation (or full-wave retardation) and light at the other wavelength, where the BPs do not have full-wave retardation any more, suffers loss at the output polarizer. Its transmittance is given by |sin2*θ*cos(Γ/2)sin*Mχ*/sin*χ*|^2^ where cos*χ* = cos2*θ*cos(Γ/2), and its complemented transmittance becomes [1 − |sin2*θ*cos(Γ/2)sin*Mχ*/sin*χ*|^2^].

2 $${t}_{f}=\frac{1}{4}[3-2\,\cos ({\rm{\Gamma }}+\varphi )-{\cos }^{2}({\rm{\Gamma }}+\varphi )]$$

**Figure 2:**
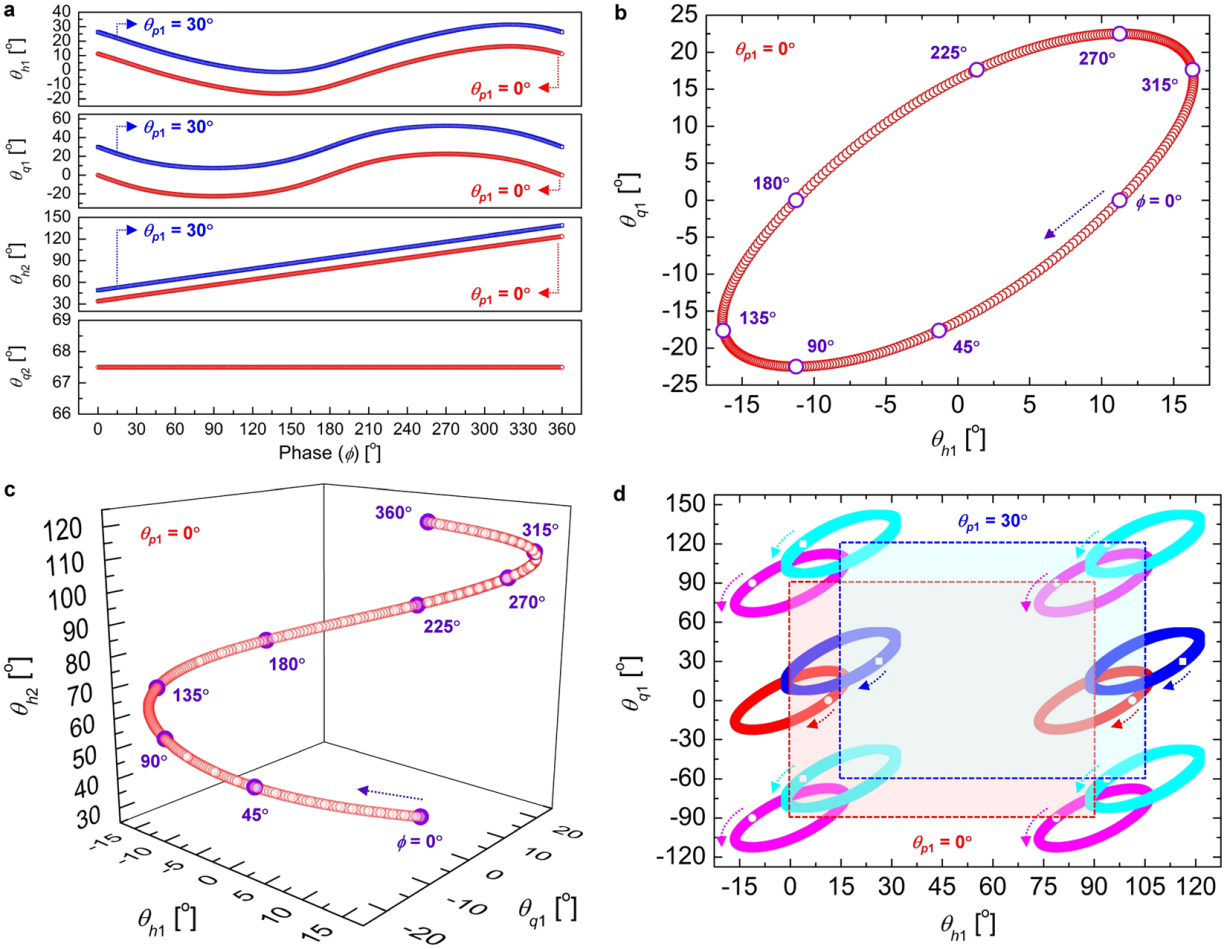
(**a**) Four waveplate angles *θ*_*h*1_, *θ*_*q*1_, *θ*_*h*2_, and *θ*_*q*2_ as a function of phase shift *ϕ* for wavelength tuning of flat-top transmittance function at *θ*_*p*1_ = 0° (red circles) and *θ*_*p*1_ = 30° (blue squares). These angle sets of (*θ*_*h*1_, *θ*_*q*1_, *θ*_*h*2_, *θ*_*q*2_) are obtained for *ϕ* increasing from 0° to 360° with a step of 1°. Loci of (**b**) (*θ*_*h*1_, *θ*_*q*1_) and (**c**) (*θ*_*h*1_, *θ*_*q*1_, *θ*_*h*2_) at *θ*_*p*1_ = 0° in Cartesian coordinate system. (**d**) Periodicity of waveplate angle loci of (*θ*_*h*1_, *θ*_*q*1_) for two cases of *θ*_*p*1_ = 0° and *θ*_*p*1_ = 30°. There are two types of (*θ*_*h*1_, *θ*_*q*1_) loci, i.e., those with CW and CCW orbits, displayed as red and magenta circles for *θ*_*p*1_ = 0° and blue and sky-blue squares for *θ*_*p*1_ = 30°, respectively. Open (white) symbols (circles and squares) in these loci indicate starting points of orbits where *ϕ* = 0°.

In order to examine the continuous frequency tunability of the filter, the orientation angle sets of the four waveplates, (*θ*_*h*1_, *θ*_*q*1_, *θ*_*h*2_, *θ*_*q*2_), which can induce an arbitrary phase shift *ϕ* ranging from 0 (0°) to 2*π* (360°) in the flat-top transmittance function, are investigated using (1) and (2). Figure 2(a) shows four waveplate angles *θ*_*h*1_, *θ*_*q*1_, *θ*_*h*2_, and *θ*_*q*2_ as a function of the phase shift *ϕ* for the wavelength tuning of the flat-top transmittance function shown in (2). These angle sets of (*θ*_*h*1_, *θ*_*q*1_, *θ*_*h*2_, *θ*_*q*2_) are obtained for *ϕ* increasing from 0° to 360° with a step of 1°. When *ϕ* changes from 0° to 360°, the variations of *θ*_*h*1_ and *θ*_*q*1_ are wave-like (but, not sinusoidal) curves, and those of *θ*_*h*2_ and *θ*_*q*2_ are linear curves. It is natural that these angle variations with *ϕ* directly depend on the slow-axis orientation angle of PMF 1, or *θ*_*p*1_. At *θ*_*p*1_ = 0° (red circles), *θ*_*h*1_ and *θ*_*q*1_ alternate over *ϕ*, being bounded in the following ranges: −16.5° < *θ*_*h*1_ < 16.5° and −22.5° ≤ *θ*_*q*1_ ≤ 22.5°. *θ*_*h*2_ monotonically increases from 33.75° to 123.75°. Similarly, at *θ*_*p*1_ = 30° (blue squares), −1.4° < *θ*_*h*1_ < 31.4°, 7.5° ≤ *θ*_*q*1_ ≤ 52.5°, and 48.75° ≤ *θ*_*h*2_ ≤ 138.75°. In particular, *θ*_*q*2_ is fixed as 67.5° in both cases. Figure 2(b) and (c) show the loci of (*θ*_*h*1_, *θ*_*q*1_) and (*θ*_*h*1_, *θ*_*q*1_, *θ*_*h*2_) at *θ*_*p*1_ = 0°, which look like a Lissajous pattern and an oval spiral in the Cartesian coordinate system, respectively. With the increase of *ϕ*, the point (*θ*_*h*1_, *θ*_*q*1_) on the elliptical locus in Fig. 2(b) travels CW along the locus. This elliptical locus of (*θ*_*h*1_, *θ*_*q*1_) is determined by the following relations (3) and (4) whose derivation can be found in part 2 of Supplementary Information online.

3 $$\sin \,2(2{\theta }_{h1}-{\theta }_{q1})=\frac{1}{\sqrt{2}}\,\cos \,\varphi $$

4 $$\tan \,2{\theta }_{q1}=\frac{\sin \,2{\theta }_{p1}-\,\cos \,2{\theta }_{p1}\,\sin \,\varphi }{\cos \,2{\theta }_{p1}+\,\sin \,2{\theta }_{p1}\,\sin \,\varphi }$$

The point (*θ*_*h*1_, *θ*_*q*1_, *θ*_*h*2_) on the helical locus in Fig. 2(c), whose projection on the *θ*_*h*1_−*θ*_*q*1_ plane is equal to the locus in Fig. 2(b), also moves from bottom to top with increasing *ϕ*. These plain and deterministic locus patterns can facilitate the prediction of the waveplate angles for the continuous wavelength tuning of filter spectra.

Figure 2(d) shows the periodicity of the waveplate angle loci of (*θ*_*h*1_, *θ*_*q*1_) for the continuous tuning for two cases of *θ*_*p*1_ = 0° and *θ*_*p*1_ = 30°. As can be seen from the figure, there are two types of elliptical loci, i.e., those with CW and CCW orbits, displayed as red and magenta circles for *θ*_*p*1_ = 0° and blue and sky-blue squares for *θ*_*p*1_ = 30°, respectively. Open (white) symbols (circles and squares) in these loci indicate starting points of orbits where *ϕ* = 0°. Each type has a period of 90° and 180° for *θ*_*h*1_ and *θ*_*q*1_, respectively. A dashed rectangle implies the minimum unit that can be allocated in a two-dimensional (*θ*_*h*1_, *θ*_*q*1_) frame satisfying this periodicity, like a crystallographic unit cell. One unit rectangle effectively contains two elliptical loci, each of which has different orbit direction. At *θ*_*p*1_ = 0°, indicated as a red-dashed box, this unit rectangle has four vertices of (0°, −90°), (90°, −90°), (90°, 90°), and (0°, 90°). In comparison with the case of *θ*_*p*1_ = 0°, the unit rectangle at *θ*_*p*1_ = 30° shifts by 15° and 30° toward + *θ*_*h*1_ and + *θ*_*q*1_ axes, respectively. It is clearly seen from Fig. 2 that a waveplate angle set (*θ*_*h*1_, *θ*_*q*1_, *θ*_*h*2_, *θ*_*q*2_) always exists for *ϕ* that increases from 0° to 360° with a step of 1° although there is not necessarily a one-to-one correspondence between (*θ*_*h*1_, *θ*_*q*1_, *θ*_*h*2_, *θ*_*q*2_) and *ϕ*. Even for *ϕ* with a finer step < 1°, the same traces are obtained in each figure. This proves that the WPC of the proposed filter enables the continuous wavelength tuning of the flat-top transmittance function.

**Figure 3:**
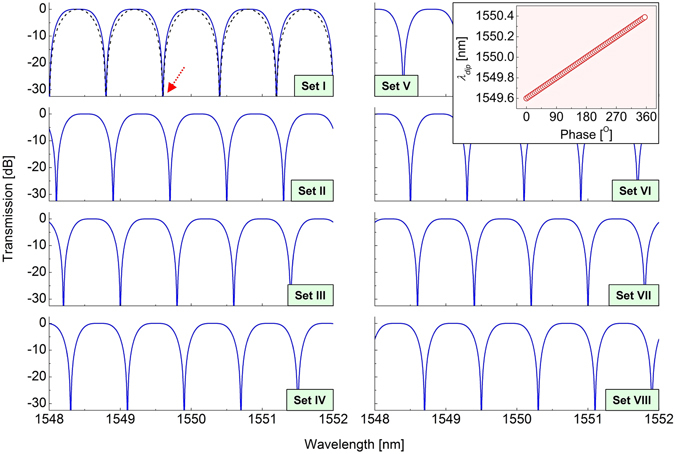
Calculated passband-flattened transmission spectra obtained at eight waveplate angle sets (Sets I–VIII) in Table 1. In this spectra, the length *L* and birefringence *B* of each PMF were set as 7.2 m and 4.166 × 10^−4^ to achieve an FSR of ~0.8 nm at 1550 nm, respectively. A black dashed line displays the zeroth-order comb spectrum for comparison. The inset shows the variation of *λ*_*dip*_ (indicated by a red arrow at Set I) for waveplate angle sets that provide finer values of *ϕ* (step: 1°).

Table 1 shows the eight selected sets of the waveplate angles (*θ*_*h*1_, *θ*_*q*1_, *θ*_*h*2_) for the wavelength tuning, which are designated as Sets I, II, III, IV, V, VI, VII, and VIII, and corresponding flat-top transmittances of the filter. These eight sets allow *ϕ* to take the values of 0°, 45°, 90°, 135°, 180°, 225°, 270°, and 315°, which are also indicated in Fig. 2(b) and (c). In other words, the flat-top comb spectrum of the filter is red-shifted by *π*/4, *π*/2, 3*π*/4, *π*, 5*π*/4, 3*π*/2, and 7*π*/4 at Sets II, III, IV, V, VI, VII, and VIII, respectively, compared with the spectrum obtained by (3−2cosΓ−cos^2^Γ)/4 at Set I. This kind of waveplate angle sets for the wavelength tuning can also be obtained for *θ*_*q*2_ = 157.5° at all Sets I−VIII like the case of *θ*_*q*2_ = 67.5° (see Supplementary Table S1 and part 3 of Supplementary Information). Moreover, at Sets III and VII, waveplate angle sets (*θ*_*h*1_, *θ*_*q*1_, *θ*_*h*2_) to obtain corresponding transmittances, i.e., [3−2cos(Γ + *π*/2)−cos^2^(Γ + *π*/2)]/4 and [3−2cos(Γ + 3*π*/2)−cos^2^(Γ + 3*π*/2)]/4, respectively, always exist for any value of *θ*_*q*2_. Except for these eight selected sets, other waveplate angle sets for different *ϕ* can be effortlessly found based on (3) and (4). Figure 3 shows the calculated passband-flattened transmission spectra obtained at the eight selected waveplate angle sets (Sets I−VIII) in Table 1. In these theoretical spectra, the length *L* and birefringence *B* of each PMF were set as 7.2 m and 4.166 × 10^–4^ to achieve an FSR of ~0.8 nm at 1550 nm, respectively. It can be found from the figure that the passband-flattened comb spectrum, compared with the zeroth-order comb spectrum indicated as a black dashed line, moves toward a longer wavelength region while the waveplate angle set changes from Set I to Set VIII. At Set I, one of transmission minima is located at *λ*_*dip*_ = 1549.6 nm, indicated as a red arrow. From Set II to Set VIII, this dip wavelength (*λ*_*dip*_) increases by 0.1 nm per waveplate angle set, ending up with 1550.3 nm. The inset shows the variation of *λ*_*dip*_ for waveplate angle sets that provide finer values of *ϕ* (step: 1°). Dense circular symbols and their linear *λ*_*dip*_−*ϕ* behavior indicate the continuous wavelength tuning capability of the filter. Consequently, it is clearly seen that the proposed filter can offer full and continuous wavelength tunability within the FSR through the proper selection of the waveplate angles (*θ*_*h*1_, *θ*_*q*1_, *θ*_*h*2_, *θ*_*q*2_). The wavelength tuning step is directly determined by the angular resolution of the rotatable waveplates. If we restrict this discussion to *θ*_*h*2_ only and the angular resolution of the waveplate is assumed as 1°, the theoretical tuning step becomes ~0.0089 nm, or ~1.11 GHz for a spectral shift of 0.1 nm during the adjustment of *θ*_*h*2_ by 11.25°.

**Table 1:** Eight selected sets of waveplate angles for wavelength tuning and corresponding filter transmittances with flat-top passbands.

	Waveplate orientation angle sets (*θ*_*h*1_, *θ*_*q*1_, *θ*_*h*2_)			Transmittance
	*θ*_*h*1_	*θ*_*q*1_	*θ*_*h*2_	
Set I	*θ*_*p*1_/2 + 11.25°	*θ*_*p*1_	*θ*_*p*1_/2 + 33.75°	(3−2cosΓ−cos^2^Γ)/4
Set II	*θ*_*p*1_/2 + [3tan^−1^(2^1/2^)/*π* −1]15°	*θ*_*p*1_ + [2tan^−1^(2^1/2^)/*π*−1]45°	*θ*_*p*1_/2 + 45°	[3−2cos(Γ + *π*/4)−cos^2^(Γ + *π*/4)]/4
Set III	*θ*_*p*1_/2−11.25°	*θ*_*p*1_−22.5°	*θ*_*p*1_/2 + 56.25°	[3−2cos(Γ + *π*/2)−cos^2^(Γ + *π*/2)]/4
Set IV	*θ*_*p*1_/2 + [3tan^−1^(2^1/2^)/*π*−2]15°	*θ*_*p*1_ + [2tan^−1^(2^1/2^)/*π*−1]45°	*θ*_*p*1_/2 + 67.5°	[3−2cos(Γ + 3*π*/4)−cos^2^(Γ + 3*π*/4)]/4
Set V	*θ*_*p*1_/2−11.25°	*θ*_*p*1_	*θ*_*p*1_/2 + 78.75°	[3−2cos(Γ + *π*)−cos^2^(Γ + *π*)]/4
Set VI	*θ*_*p*1_/2−[3tan^−1^(2^1/2^)/*π* −1]15°	*θ*_*p*1_−[2tan^−1^(2^1/2^)/*π*−1]45°	*θ*_*p*1_/2 + 90°	[3−2cos(Γ + 5*π*/4)−cos^2^(Γ + 5*π*/4)]/4
Set VII	*θ*_*p*1_/2 + 11.25°	*θ*_*p*1_ + 22.5°	*θ*_*p*1_/2 + 101.25°	[3−2cos(Γ + 3*π*/2)−cos^2^(Γ + 3*π*/2)]/4
Set VIII	*θ*_*p*1_/2−[3tan^−1^(2^1/2^)/*π*−2]15°	*θ*_*p*1_−[2tan^−1^(2^1/2^)/*π*−1]45°	*θ*_*p*1_/2 + 112.5°	[3−2cos(Γ + 7*π*/4)−cos^2^(Γ + 7*π*/4)]/4

## Experimental results

**Figure 4:**
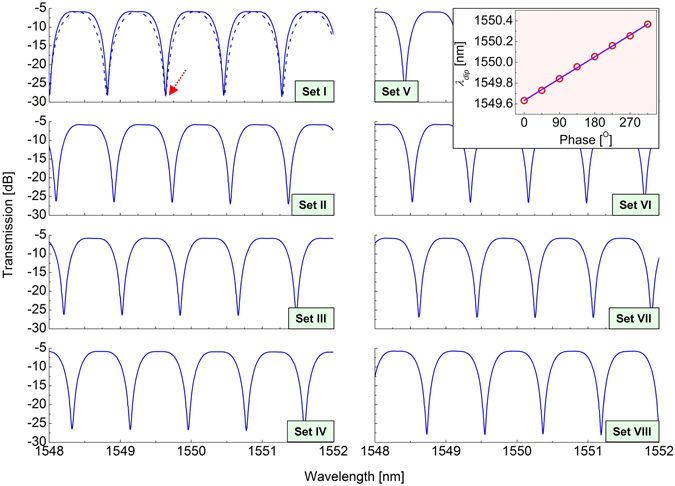
Measured wavelength-tuned transmission spectra at eight waveplate angle sets (Sets I-VIII) in Table 2. A black dashed curve displays the measured zeroth-order comb spectrum for comparison. The inset shows the wavelength shift of *λ*_*dip*_ (indicated by a red arrow at Set I), obtained at the eight angle sets. Linear regression resulted in an adjusted *R* ^2^ value of ~0.99948.

For the experimental demonstration of the filter wavelength tunability, the proposed filter was fabricated by utilizing a fiber-pigtailed four-port PBS (OZ Optics), two fiber-pigtailed HWPs (OZ Optics), two fiber-pigtailed QWPs (OZ Optics), and two equal-length bow-tie type PMF segments (Fibercore). As shown in Fig. 1, two BE groups, each of which comprised of an HWP, a QWP, and one PMF segment, were constructed. One end of PMF in the second BE group, or PMF 2, was butt-coupled to port R of the PBS so that the slow axis of PMF 2 should be oriented at 22.5° with respect to the *x* axis. The other end of PMF 2 was connected to the QWP in the second BE group, or QWP 2. The length of each PMF whose birefringence was ~4.166 × 10^−4^ was ~7.07 m, and the FSR was measured as ~0.816 nm around 1550 nm. An amplified spontaneous emission source (Fiberlabs FL7701) and an optical spectrum analyzer (Yokogawa AQ6370C) were employed to measure the transmission spectra of the filter. Figure 4 shows wavelength-tuned transmission spectra measured at the eight waveplate angle sets (Sets I–VIII) shown in Table 2, and a black dashed curve indicates the zeroth-order comb spectrum for comparison. As depicted in the theoretical prediction, passband-flattened comb spectra shift by ~0.1 nm toward a longer wavelength region each time the angle set is changed to an adjacent set, e.g., from Set III to Set IV. The inset of Fig. 4 shows the wavelength shift of *λ*_*dip*_ (indicated as a red arrow), obtained at the eight angle sets. The coefficient of determination *R* ^*2*^ for the linear regression was estimated as a value of ~0.99948, which is closest to unity, showing considerably linear relationship between applied phase shift *ϕ* and *λ*_*dip*_. It was also experimentally checked that any arbitrary phase shifts of 0°−360° except for the eight discrete ones could be introduced into the filter transmittance by selecting proper waveplate angles. In other words, the passband of the filter could be moved into a desired wavelength location. As a result, it is concluded that the appropriate selection of (*θ*_*h*1_, *θ*_*q*1_, *θ*_*h*2_, *θ*_*q*2_) can allow the filter to provide full and continuous wavelength tunability.

**Table 2:** Eight selected sets of waveplate angles for implementation of filter wavelength tuning.

	Waveplate orientation angle sets (*θ*_*h*1_, *θ*_*q*1_, *θ*_*h*2_, *θ*_*q*2_)			
	*θ*_*h*1_	*θ*_*q*1_	*θ*_*h*2_	*θ*_*q*2_
Set I	65°	73°	48°	72°
Set II	64°	68°	68°	72°
Set III	78°	68°	78°	72°
Set IV	90°	73°	84°	70°
Set V	101°	118°	16°	70°
Set VI	93°	114°	28°	70°
Set VII	79°	104°	38°	70°
Set VIII	59°	67°	49°	70°

The IL of the filter passband was measured as ~5.87 dB, which was mainly caused by losses from the PBS itself (including fiber coupling loss), the butt-coupling between PMF 2 and the PBS, four waveplates, and fusion splicing between PMF and single-mode fiber (SMF) used for waveplate pigtails. In particular, the average passband flatness within one channel was measured as ~0.058 dB. The 3 dB bandwidth of the passband increased by ~28.7% in comparison with that of the zeroth-order comb filter (see Supplementary Fig. S2(a) and part 4 of Supplementary Information). The spectral flatness between multiple channels was measured as ~0.034 dB at 1548−1552 nm, but increased to ~0.244 dB at a relatively wider wavelength range from 1542 to 1558 nm. And the ER of the filter was over ~20.12 dB at the above 16 nm range (see Supplementary Fig. S2(b) and part 4 of Supplementary Information). Waveplate angle deviations between theoretical and experimental sets in the tables originate from the weak but inherent birefringence of SMF used for connecting the PBS, waveplates, and PMF segments. This SMF birefringence can vary the SOP of light passing through the filter differently than originally expected in the propagation paths in Fig. 1(b) ^32^. Moreover, the polarization dependence of the fabricated filter was also examined by utilizing another polarization controller (Agilent 8169 A), positioned in front of port 1 of the PBS. The maximum polarization sensitivity was less than 0.5 dB, which may be attributed to the polarization-dependent loss of a photodetector in the OSA and imperfection in the splitting ratio of the PBS. In addition, the wavelength tuning speed, directly affected by the setting speed of the rotatable waveplates, can reach sub microseconds if electrically controllable waveplates are incorporated.

### Relationship between wavelength tuning and SOP evolution within filter

**Figure 5:**
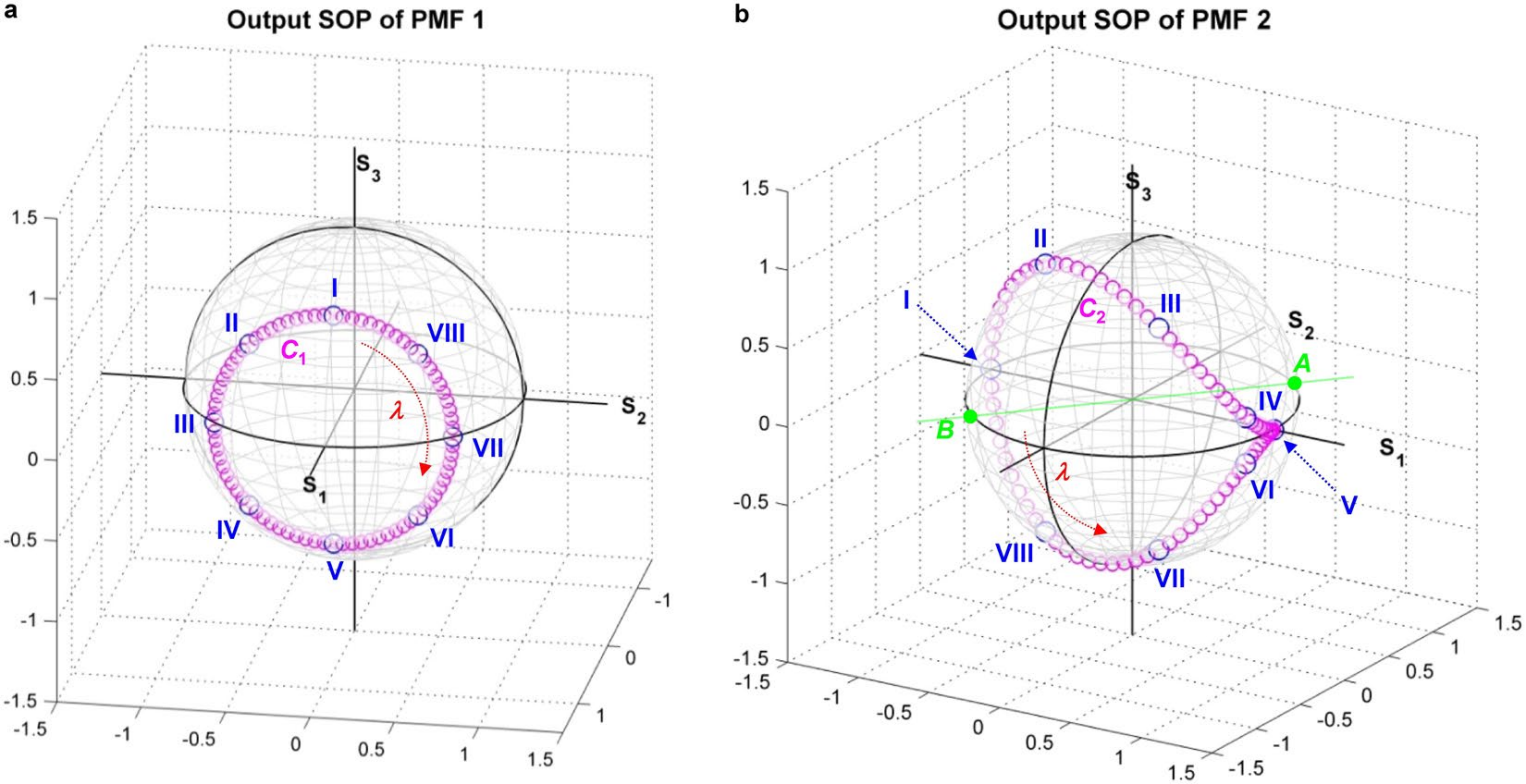
(**a**) Spectral evolution of SOP_out_ of PMF 1 within FSR Δ*λ*_*s*_ in CW path for Sets I–VIII in Table 1 at *θ*_*p*1_ = 0°. As indicated by a red arrow, the SOP_out_ traces out a circle *C*_1_ on the Poincare sphere as the wavelength increases from *λ*_*A*_ to *λ*_*B*_, moving CW around the *S*_1_ axis. (**b**) Spectral evolution of SOP_out_ of PMF 2 within FSR Δ*λ*_*s*_ in CW path for Sets I–VIII in Table 1. Similarly, as indicated by a red arrow, the SOP_out_ makes one CW rotation around the *S*_2_ axis along the trajectory *C*_2_ of a droplet shape with increasing wavelength.

Finally, in order to explore the relationship between the wavelength tuning and the spectral evolution of the SOP of light propagating through the filter, the wavelength-dependent evolution of output SOPs (SOP_out_’s) of PMF 1 and PMF 2 are investigated in terms of the Poincare sphere at the eight waveplate angle sets (Sets I−VIII) of Table 1. Figure 5(a) shows the spectral evolution of the SOP_out_ of PMF 1 within the FSR Δ*λ*_*s*_ in the CW path for Sets I–VIII at *θ*_*p*1_ = 0°. As indicated by a red arrow, the SOP_out_ traces out a circle *C*_1_, which has a radius of (1/2)^1/2^ and a center of (2*ε* = 0°, 2*ψ* = 0°), on the Poincare sphere as the wavelength increases. Here, 2*ε* (−90° ≤ 2*ε* ≤ 90°) and 2*ψ* (−180° ≤ 2*ψ* ≤ 180°) are the latitude and longitude of the Poincare sphere, respectively. When the wavelength increases from *λ*_*A*_ to *λ*_*B*_ where *λ*_*B*_ − *λ*_*A*_ = Δ*λ*_*s*_, this SOP_out_ makes one CW revolution around the *S*_1_ axis on the Poincare sphere. The initial point *P*_1_ of this revolution (at *λ*_*A*_), indicated as a blue open circle, is directly dependent on the SOP_in_ of PMF 1, that is, is determined by the orientation angles of HWP 1 and QWP 1, *θ*_*h*1_ and *θ*_*q*1_. Actually, *P*_1_ also indicates the SOP_in_ of PMF 1. For example, *P*_1_ on *C*_1_ is displayed as I to VIII when the waveplate angle set is selected as from Set I to Set VIII in Table 1. At other *θ*_*p*1_ (≠0°), the center of the circle *C*_1_ is shifted by 2*ψ* = 2*θ*_*p*1_ and becomes (2*ε* = 0°, 2*ψ* = 2*θ*_*p*1_), but the spectral behavior of the SOP_out_ is identical to the case of *θ*_*p*1_ = 0°. The WPC of HWP 1 and QWP 1 plays a role of defining the radius of this circular trace, centered at one point on the equator of the Poincare sphere, and *P*_1_ on this trace. The size and shape of this trace determine those of the trajectory formed by the SOP_out_’s of PMF 2.

Figure 5(b) shows the spectral evolution of the SOP_out_ of PMF 2 within the FSR Δ*λ*_*s*_ in the CW path for Sets I−VIII. As indicated by a red arrow, the SOP_out_ makes one CW rotation around the *S*_2_ axis along the trajectory *C*_2_ of a droplet shape with the increase of wavelength from *λ*_*A*_ to *λ*_*B*_. The same trajectory is obtained for any *θ*_*p*1_ because *θ*_*p*2_ is fixed as 22.5°. While the waveplate angle set changes from Set I to Set VIII, the initial point *P*_2_ of the spectral evolution (at *λ*_*A*_) on *C*_2_ varies from I to VIII, moving CCW around the *S*_2_ axis on the sphere. Similarly, *P*_2_ on *C*_2_, indicated as a blue open circle, directly depends on the SOP_in_ of PMF 2, which is determined by *θ*_*h*1_, *θ*_*q*1_, *θ*_*h*2_, and *θ*_*q*2_. In this case though, this SOP_in_ is not independent of wavelength. Over a wavelength span of Δ*λ*_*s*_, the SOP_in_ of PMF 2 has a circular trace, whose radius is (1/2)^1/2^ and plane is parallel to a straight line *AB* connecting two points, *A* (2*ε* = 0°, 2*ψ* = 45°) and *B* (2*ε* = 0°, 2*ψ* = −135°) on the Poincare sphere, indicated as a solid green line (see Supplementary Videos S1–S4 and part 5 of Supplementary Information).

For obtaining this spectrally evolving circular trace (SOP_in_ of PMF 2), the WPC of HWP 2 and QWP 2 renders the circular trajectory of the SOP_out_ of PMF 1, shown in Fig. 5(a), to move onto a plane that is distant by (1/2)^1/2^ from the line *AB*. The initial point *P*_*in*_ of the spectral evolution on this SOP_in_ trace, which is already determined by *θ*_*h*1_ and *θ*_*q*1_, becomes the initial point *P*_2_ of the spectral evolution on the droplet-shaped trajectory *C*_2_ of the SOP_out_ of PMF 2 (see Supplementary Videos S1–S4 and part 5 of Supplementary Information). For example, *P*_*in*_ on an SOP_in_ circular trace centered at (2*ε* = 0°, 2*ψ* = 135°) should be located at I on *C*_2_ at Set I. At Set II, *P*_*in*_ on an SOP_in_ circular trace centered at (2*ε* = 45°, 2*ψ* = 135°) should move onto II on *C*_2_. If *P*_*in*_ is outside *C*_2_ (possibly mediated by other *θ*_*h*1_ and *θ*_*q*1_ beyond the angle sets in Fig. 2(a)) or if this SOP_in_ trace is not located at a position separated by (1/2)^1/2^ in parallel with the line *AB* (possibly mediated by other *θ*_*h*2_ and *θ*_*q*2_ beyond the angle sets in Fig. 2(a)), the SOP_out_ trajectory does not appear at the same position as *C*_2_ shown in Fig. 5(b) although its shape and size remain the same. At this situation of the angular deviation, the same flat-top transmittance as Fig. 3 is not obtained. On behalf of the continuous wavelength tuning of the flat-top transmittance for a Δ*λ*_*s*_ span, therefore, the SOP_in_ circular trace over Δ*λ*_*s*_, which is initially centered at (2*ε* = 0°, 2*ψ* = 135°) at Set I, should make one CCW revolution around the line *AB*. Simultaneously, it should be also satisfied that the SOP_in_ of PMF 2 at *λ*_*A*_, or *P*_*in*_, moves CCW along *C*_2_ around the *S*_2_ axis according to the above revolution of the SOP_in_ circular trace, starting from I at Set I. In simple terms, this SOP_in_ circular trace should both revolve around the line *AB* and rotate itself around its center (see Supplementary Videos S1–S4 and part 5 of Supplementary Information).

At Set I, while the wavelength increases from *λ*_*A*_ to *λ*_*A*_ + 7Δ*λ*_*s*_/8, the SOP_out_ of PMF 2 evolves from I to II (via VIII, VII, VI, V, IV, and III) moving CW around the *S*_2_ axis, as shown in Fig. 5(b). In this case, the passband dip and center stand at *λ*_*dip*_ = *λ*_*A*_ and *λ*_*center*_ = *λ*_*A*_ + Δ*λ*_*s*_/2, respectively, because the SOP_out_ of PMF 2 before the horizontal analyzer becomes LVP at *λ*_*A*_ and LHP at *λ*_*A*_ + Δ*λ*_*s*_/2, which correspond to points I and V, respectively. At Set II, the initial point of spectral evolution on the SOP_out_ trajectory changes into II with the final SOP_out_ ending up with III at the same wavelength span (from *λ*_*A*_ to *λ*_*A*_ + 7Δ*λ*_*s*_/8). This SOP_out_ evolution makes *λ*_*dip*_ and *λ*_*center*_ shift to *λ*_*A*_ + Δ*λ*_*s*_/8 and *λ*_*A*_ + 5Δ*λ*_*s*_/8, respectively. For other remaining cases (Sets III–VIII), the initial SOP_out_ changes from III to VIII, and similar spectral evolution of the SOP_out_ is made with the increase of wavelength from *λ*_*A*_ to *λ*_*A*_ + 7Δ*λ*_*s*_/8. While the waveplate angle set progresses from Set III to Set VIII, *λ*_*center*_ and *λ*_*dip*_ shift by Δ*λ*_*s*_/8 per set. In brief, Sets I–VIII in Table 1 allow *ϕ* in (2) to be 0°, 45°, 90°, 135°, 180°, 215°, 270°, and 315°, in sequence, and the flat-top transmittance function red-shifts totally by 7Δ*λ*_*s*_/8 during the transition from Set I to Set VIII. In other words, if (*θ*_*h*1_, *θ*_*q*1_, *θ*_*h*2_, *θ*_*q*2_) are properly chosen, the passband-flattened spectrum of the filter can be continuously frequency-shifted over a full wavelength range.

## Conclusion

In conclusion, we demonstrated a continuously tunable polarization-independent passband-flattened fiber comb filter by incorporating a polarization-diversified loop comprised of a PBS, two PMF segments, and four waveplates (two HWPs and two QWPs). First, the filter transmittance was derived using Jones matrix formulation. Second, orientation angle sets of the four waveplates, which can induce an arbitrary phase shift from 0 to 2*π* in the flat-top transmittance, were found on the basis of the derived transmittance. It was confirmed from theoretical transmission spectra obtained at some selected waveplate angle sets that the passband-flattened comb spectrum could be continuously frequency-tuned by properly controlling the four waveplates. Then, the theoretical prediction was verified by experiments. In the fabricated filter, the absolute wavelength position of the flat-top transmission spectrum could be continuously shifted by the appropriate choice of the waveplate angles. In particular, the spectral evolution of the SOP_out_ of each PMF segment in the filter was investigated on the Poincare sphere for the selected waveplate angle sets. And the relationship between the continuous wavelength tuning and the SOP evolution within the filter were also revealed. The continuous tuning feature of our filter can be beneficially utilized in various applications including microwave and optical signal processing, waveband switching in multi-granular networks, multiwavelength lasing, and optical sensor demodulation.

### Data Availability

All data generated or analysed during this study are included in this published article (and its Supplementary Information files).

## Electronic supplementary material

Supplementary Information

Supplementary Video 1

Supplementary Video 2

Supplementary Video 3

Supplementary Video 4
