# Supplementary material for: Continuously wavelength-tunable passband-flattened fiber comb filter based on polarization-diversified loop structure
Source: Sci Rep. 2017 Aug 16;7:8311. doi: 10.1038/s41598-017-06952-z (PMC5559556; doi:10.1038/s41598-017-06952-z)
Supplement: Supplementary file 1 — Supplementary Information [file 41598_2017_6952_MOESM1_ESM.doc]

**Supplementary Information**

Continuously wavelength-tunable passband-flattened fiber comb filter based on polarization-diversified loop structure

Jaehoon Jung^1^ and Yong Wook Lee^2,*^

^1^Department of Electronics and Electrical Engineering, Dankook University, Gyeonggi-do, Yongin 16890, South Korea

^2^Interdisciplinary Program of Biomedical Mechanical & Electrical Engineering and School of Electrical Engineering, Pukyong National University, Yongso-ro 45, Nam-Gu, Busan 48513, South Korea

*Corresponding author’s E-mail: yongwook@pknu.ac.kr

**Part 1. Detailed expression of transfer matrices of filter, PMF, and waveplates**

The detailed expression on *T* and the transfer matrices of PMF and waveplates are given as follows.

$$T=\left[\begin{matrix} 1 & 0 \\ 0 & 0 \end{matrix}\right]T_{PMF2}\left(22.5^{\circ}\right)T_{QWP2}\left(\theta_{q2}\right)T_{HWP2}\left(\theta_{h2}\right)T_{PMF1}\left(\theta_{p1}\right)T_{QWP1}\left(\theta_{q1}\right)T_{HWP1}\left(\theta_{h1}\right)\left[\begin{matrix} 1 & 0 \\ 0 & 0 \end{matrix}\right]$$

$$+\left[\begin{matrix} 0 & 0 \\ 0 & 1 \end{matrix}\right]T_{HWP1}\left(-\theta_{h1}\right)T_{QWP1}\left(-\theta_{q1}\right)T_{PMF1}\left(-\theta_{p1}\right)T_{HWP2}\left(-\theta_{h2}\right)T_{QWP2}\left(-\theta_{q2}\right)T_{PMF2}\left(-22.5^{\circ}\right)\left[\begin{matrix} 0 & 0 \\ 0 & 1 \end{matrix}\right]$$

where *T_HWP_*_1_, *T_QWP_*_1_, *T_PMF_*_1_, *T_HWP_*_2_, *T_QWP_*_2_, and *T_PMF_*_2_ are the Jones matrices of HWP 1, QWP 1, PMF 1, HWP 2, QWP 2, and PMF 2 that have slow-axis orientation angles of *θ_h_*_1_, *θ_q_*_1_, *θ_p_*_1_, *θ_h_*_2_, *θ_q_*_2_, and 22.5° with respect to the *x* axis, respectively.

$\begin{matrix} T_{HWP1}=R\left(-\theta_{h1}\right)\left[\begin{matrix} e^{-j\frac{\pi}{2}} & 0 \\ 0 & e^{j\frac{\pi}{2}} \end{matrix}\right]R\left(\theta_{h1}\right)=-j\left[\begin{matrix} \cos2\theta_{h1} & \sin2\theta_{h1} \\ \sin2\theta_{h1} & -\cos2\theta_{h1} \end{matrix}\right]\text{ }\left(\text{where }R\left(\theta\right)=\left[\begin{matrix} \cos\theta& \sin\theta\\ -\sin\theta& \cos\theta\end{matrix}\right]\right) \\ \\ T_{HWP2}=R\left(-\theta_{h2}\right)\left[\begin{matrix} e^{-j\frac{\pi}{2}} & 0 \\ 0 & e^{j\frac{\pi}{2}} \end{matrix}\right]R\left(\theta_{h2}\right)=-j\left[\begin{matrix} \cos2\theta_{h2} & \sin2\theta_{h2} \\ \sin2\theta_{h2} & -\cos2\theta_{h2} \end{matrix}\right] \\ \\ T_{QWP1}=R\left(-\theta_{q1}\right)\left[\begin{matrix} e^{-j\frac{\pi}{4}} & 0 \\ 0 & e^{j\frac{\pi}{4}} \end{matrix}\right]R\left(\theta_{q1}\right)=\frac{\sqrt{2}}{2}\left[\begin{matrix} 1-j\cos2\theta_{q1} & -j\sin2\theta_{q1} \\ -j\sin2\theta_{q1} & 1+j\cos2\theta_{q1} \end{matrix}\right] \\ \\ T_{QWP2}=R\left(-\theta_{q2}\right)\left[\begin{matrix} e^{-j\frac{\pi}{4}} & 0 \\ 0 & e^{j\frac{\pi}{4}} \end{matrix}\right]R\left(\theta_{q2}\right)=\frac{\sqrt{2}}{2}\left[\begin{matrix} 1-j\cos2\theta_{q2} & -j\sin2\theta_{q2} \\ -j\sin2\theta_{q2} & 1+j\cos2\theta_{q2} \end{matrix}\right] \end{matrix}$

$T_{PMF1}=R\left(-\theta_{p1}\right)\left[\begin{matrix} e^{-j\frac{\Gamma}{2}} & 0 \\ 0 & e^{j\frac{\Gamma}{2}} \end{matrix}\right]R\left(\theta_{p1}\right)=\left[\begin{matrix} \cos\left(\frac{\Gamma}{2}\right)-j\cos2\theta_{p1}\sin\left(\frac{\Gamma}{2}\right) & -j\sin2\theta_{p1}\sin\left(\frac{\Gamma}{2}\right) \\ -j\sin2\theta_{p1}\sin\left(\frac{\Gamma}{2}\right) & \cos\left(\frac{\Gamma}{2}\right)+j\cos2\theta_{p1}\sin\left(\frac{\Gamma}{2}\right) \end{matrix}\right]$

$T_{PMF2}=R\left(-22.5^{\circ}\right)\left[\begin{matrix} e^{-j\frac{\Gamma}{2}} & 0 \\ 0 & e^{j\frac{\Gamma}{2}} \end{matrix}\right]R\left(22.5^{\circ}\right)=\left[\begin{matrix} \cos\left(\frac{\Gamma}{2}\right)-j\frac{\sqrt{2}}{2}\sin\left(\frac{\Gamma}{2}\right) & -j\frac{\sqrt{2}}{2}\sin\left(\frac{\Gamma}{2}\right) \\ -j\frac{\sqrt{2}}{2}\sin\left(\frac{\Gamma}{2}\right) & \cos\left(\frac{\Gamma}{2}\right)+j\frac{\sqrt{2}}{2}\sin\left(\frac{\Gamma}{2}\right) \end{matrix}\right]$

The resulting transfer matrix *T* is represented as follows.

$\begin{matrix} T=\left[\begin{matrix} A & 0 \\ 0 & A^{*} \end{matrix}\right] \\ \\ \text{where }A=-\sin\left(2\theta_{p1}-2\theta_{h2}-\theta_{q1}+\theta_{q2}\right)\cos\left(2\theta_{h1}-\theta_{q1}+\theta_{q2}+45^{\circ}\right)\sin^{2}\left(\frac{\Gamma}{2}\right) \\ \text{ }+\left[\cos\left(2\theta_{p1}-2\theta_{h2}-\theta_{q1}+\theta_{q2}\right)\cos\left(2\theta_{h1}-\theta_{q1}-\theta_{q2}\right)\right. \\ \text{ }\left.\text{+}\cos\left(2\theta_{h2}-\theta_{q1}-\theta_{q2}\right)\sin\left(2\theta_{h1}-\theta_{q1}+\theta_{q2}+45^{\circ}\right)\right]\cos\left(\frac{\Gamma}{2}\right)\sin\left(\frac{\Gamma}{2}\right) \\ \text{ }-\sin\left(2\theta_{h1}-\theta_{q1}-\theta_{q2}\right)\sin\left(2\theta_{h2}-\theta_{q1}-\theta_{q2}\right)\cos^{2}\left(\frac{\Gamma}{2}\right) \\ \text{ }+j\cos\left(2\theta_{p1}-2\theta_{h2}-\theta_{q1}+\theta_{q2}\right)\sin\left(2\theta_{h1}-\theta_{q1}-\theta_{q2}-45^{\circ}\right)\sin^{2}\left(\frac{\Gamma}{2}\right) \\ \text{ }+\left[j\sin\left(2\theta_{p1}-2\theta_{h2}-\theta_{q1}+\theta_{q2}\right)\sin\left(2\theta_{h1}-\theta_{q1}+\theta_{q2}\right)\right.\text{ } \\ \text{ }\left.+j\sin\left(2\theta_{h2}-\theta_{q1}-\theta_{q2}\right)\cos\left(2\theta_{h1}-\theta_{q1}-\theta_{q2}-45^{\circ}\right)\right]\cos\left(\frac{\Gamma}{2}\right)\sin\left(\frac{\Gamma}{2}\right) \\ \text{ }+j\cos\left(2\theta_{h2}-\theta_{q1}-\theta_{q2}\right)\cos\left(2\theta_{h1}-\theta_{q1}+\theta_{q2}\right)\cos^{2}\left(\frac{\Gamma}{2}\right) \end{matrix}$

**Part 2. Derivation of (3) and (4)**

Let us assume that *P*_1_ is the initial point of the spectral evolution of the SOP_out_ of PMF 1 with arbitrary *θ_p_*_1_, or *C*_1_, for a certain *φ* value, as shown in Fig. S1(a). Figures S1(b) and S1(c) show the two-dimensional representation of *P*_1_ on the *S*_1_−*S*_2_ plane perpendicular to the *S*_3_ axis and on the plane parallel to the line *CD* and containing the *S*_3_ axis, respectively. Figure S(d) show the three-dimensional representation of *P*_1_. Because the SOP_out_ circular trace of PMF 1 has a diameter of 2^1/2^, the length of the line *CD* is 2^1/2^, and the lines *OO*′, *CO*′, and *DO*′ have an equal length of (1/2)^1/2^, as shown in Fig. S1(b). The distance between the projection of *P*_1_ on the *S*_1_−*S*_2_ plane, i.e., *P*_1_′ and the center of the sphere *O* is designated as *X* in convenience. The angle *S*_1_*OO*′ is 2*θ_p_*_1_ due to the slow-axis orientation angle *θ_p_*_1_ of PMF 1, and angles *P*_1_′*OS*_1_ and *P*_1_*O P*_1_′ are −2*ψ* and 2*χ* according to the Poincare sphere representation of an SOP, as shown in Figs. S1(b) and S1(d), respectively. As the angle *P*_1_*O*′*S*_3_ corresponds to *φ* in Fig. S1(c), the lengths of the lines *P*_1_′*O*′ and *P*_1_′′*O*′ become sin*φ*(1/2)^1/2^ and cos*φ*(1/2)^1/2^, respectively.

The Stokes vector of *P*_1_ is given by (1, cos2*ψ*cos2*χ*, sin2*ψ*cos2*χ*, sin2*χ*). First, sin2*χ* and cos2*χ* are cos*φ*(1/2)^1/2^ and *X*, respectively, as can be seen from Fig. S1(d). *X* can also be represented as (1−cos^2^*φ*/2)^1/2^. Then, cos(−2*ψ*+2*θ_p_*_1_) = (1/2)^1/2^/*X* = 1/(2−cos^2^*φ*)^1/2^ and sin(−2*ψ*+2*θ_p_*_1_) = sin*φ*(1/2)^1/2^/*X* = sin*φ*/(2−cos^2^*φ*)^1/2^, as can be confirmed from Figs. S1(b) and S1(c). The following equations (S1) and (S2) can be derived from these relations. Then, from these two equations, cos2*ψ* and sin2*ψ* are obtained as (cos2*θ_p_*_1_+sin2*θ_p_*_1_sin*φ*)/(2−cos^2^*φ*)^1/2^ and (sin2*θ_p_*_1_−cos2*θ_p_*_1_sin*φ*)/(2−cos^2^*φ*)^1/2^, respectively. Using sin2*χ*, cos2*χ*, sin2*ψ*, and cos2*ψ* obtained above, the Stokes vector of *P*_1_ can be reduced to (1, (cos2*θ_p_*_1_+sin2*θ_p_*_1_sin*φ*)(1/2)^1/2^, (sin2*θ_p_*_1_−cos2*θ_p_*_1_sin*φ*)(1/2)^1/2^, cos*φ*(1/2)^1/2^).


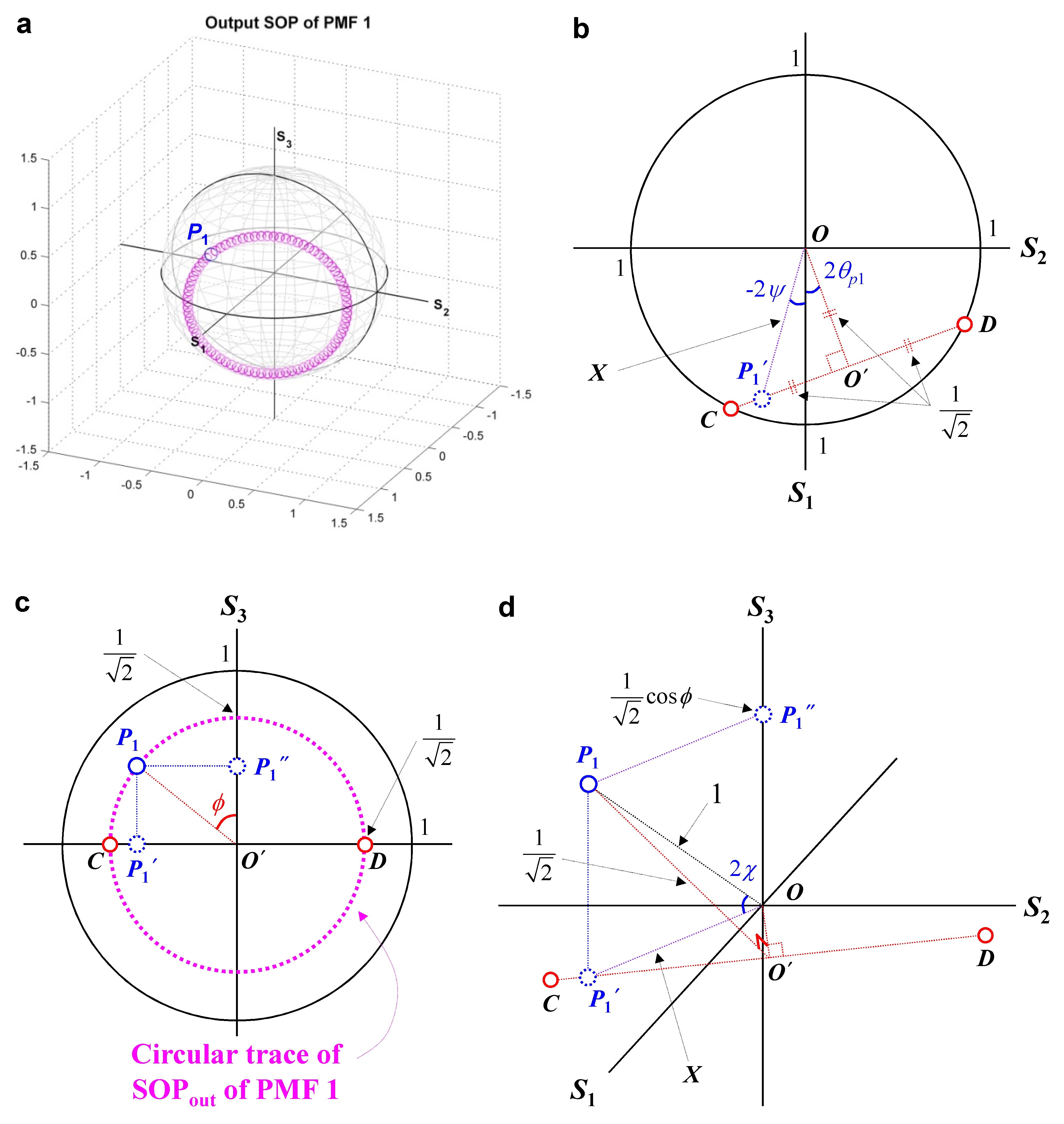


**Figure S1.** (a) Spectral evolution of SOP_out_ of PMF 1 with arbitrary *θ_p_*_1_ and its initial point *P*_1_, two-dimensional representation of *P*_1_ on (b) *S*_1_−*S*_2_ plane perpendicular to *S*_3_ axis and (c) plane parallel to line *CD* and containing *S*_3_ axis, and (d) three-dimensional representation of *P*_1_.

(S1)

(S2)

On the other hand, this Stokes vector of *P*_1_, designated as *S_P_*_1_, can also be obtained using Mueller matrices of HWP 1 and QWP 1 and is given by (S3).

$S_{P1}=M_{QWP1}\left(\theta_{q1}\right)M_{HWP1}\left(\theta_{h1}\right)S_{LHP}$ (S3)

$$\begin{matrix} \text{where }M_{QWP1}\left(\theta_{q1}\right)=\left(\begin{matrix} 1 & 0 & 0 & 0 \\ 0 & \cos^{2}2\theta_{q1} & \sin2\theta_{q1}\cos2\theta_{q1} & \sin^{2}\theta_{q1} \\ 0 & \sin2\theta_{q1}\cos2\theta_{q1} & \sin^{2}2\theta_{q1} & -\cos2\theta_{q1} \\ 0 & -\sin2\theta_{q1} & \cos2\theta_{q1} & 0 \end{matrix}\right), \\ \text{ }M_{HWP1}\left(\theta_{h1}\right)=\left(\begin{matrix} 1 & 0 & 0 & 0 \\ 0 & \cos4\theta_{h1} & \sin4\theta_{h1} & 0 \\ 0 & \sin4\theta_{h1} & -\cos4\theta_{h1} & 0 \\ 0 & 0 & 0 & -1 \end{matrix}\right),\text{ and }S_{LHP}=\left(\begin{matrix} 1 \\ 1 \\ 0 \\ 0 \end{matrix}\right) \end{matrix}$$

Here, *M_QWP_*_1_ and *M_HWP_*_1_ are Mueller matrices of QWP 1 and HWP 1, and *S_LHP_* is the Stokes vector of LHP. On the basis of (S3), *S_P_*_1_ can be reduced to (1, cos2*θ_q_*_1_cos2(2*θ_h_*_1_−*θ_q_*_1_), sin*θ_q_*_1_cos2(2*θ_h_*_1_−*θ_q_*_1_), sin2(2*θ_h_*_1_−*θ_q_*_1_)). Comparison of two Stokes vectors obtained by a graphical approach and Mueller matrices gives three following equations (S4), (S5), and (S6).

(S4)

(S5)

(S6)

Thus, (3) is directly obtained from (S6), and (4) can be derived by dividing (S5) by (S4).

**Part 3. Waveplate angle sets for wavelength tuning at QWP 2 angle other than 67.5°**

Table S1 shows eight selected sets (I−VIII) of waveplate angles for the wavelength tuning and corresponding filter transmittances with flat-top passbands for *θ_q_*_2_ = 157.5°.

| Table S1. Eight selected sets of waveplate angles for wavelength tuning  and corresponding filter transmittances with flat-top passbands for *θ_q_*_2_ = 157.5°. | | | | |
| --- | --- | --- | --- | --- |
|  | Waveplate orientation angle sets (*θ_h_*_1_, *θ_q_*_1_, *θ_h_*_2_) | | | Transmittance |
|  | *θ_h_*_1_ | *θ_q_*_1_ | *θ_h_*_2_ |  |
| Set I | *θ_p_*_1_/2−11.25° | *θ_p_*_1_ | *θ_p_*_1_/2+123.75° | (3−2cosΓ−cos^2^Γ)/4 |
| Set II | *θ_p_*_1_/2−[3tan^-1^(2^1/2^)/*π*−1]15° | *θ_p_*_1_−[2tan^-1^(2^1/2^)/*π*−1]45° | *θ_p_*_1_/2+112.5° | [3−2cos(Γ+*π*/4)−cos^2^(Γ+*π*/4)]/4 |
| Set III | *θ_p_*_1_/2+11.25° | *θ_p_*_1_+22.5° | *θ_p_*_1_/2+101.25° | [3−2cos(Γ+*π*/2)−cos^2^(Γ+*π*/2)]/4 |
| Set IV | *θ_p_*_1_/2−[3tan^-1^(2^1/2^)/*π*−2]15° | *θ_p_*_1_−[2tan^-1^(2^1/2^)/*π*−1]45° | *θ_p_*_1_/2+90° | [3−2cos(Γ+3*π*/4)−cos^2^(Γ+3*π*/4)]/4 |
| Set V | *θ_p_*_1_/2+11.25° | *θ_p_*_1_ | *θ_p_*_1_/2+78.75° | [3−2cos(Γ+*π*)−cos^2^(Γ+*π*)]/4 |
| Set VI | *θ_p_*_1_/2+[3tan^-1^(2^1/2^)/*π*−1]15° | *θ_p_*_1_+[2tan^-1^(2^1/2^)/*π*−1]45° | *θ_p_*_1_/2+67.5° | [3−2cos(Γ+5*π*/4)−cos^2^(Γ+5*π*/4)]/4 |
| Set VII | *θ_p_*_1_/2−11.25° | *θ_p_*_1_−22.5° | *θ_p_*_1_/2+56.25° | [3−2cos(Γ+3*π*/2)−cos^2^(Γ+3*π*/2)]/4 |
| Set VIII | *θ_p_*_1_/2+[3tan^-1^(2^1/2^)/*π*−2]15° | *θ_p_*_1_+[2tan^-1^(2^1/2^)/*π*−1]45° | *θ_p_*_1_/2+45° | [3−2cos(Γ+7*π*/4)−cos^2^(Γ+7*π*/4)]/4 |

**Part 4. Transmission spectra at wider and narrower wavelength ranges**

Figure S2 shows transmission spectra measured at (a) narrower (1.1 nm) and (b) wider (16 nm) wavelength ranges. A black dotted curve in Fig. S2(a) indicates the measured zeroth-order comb spectrum for comparison. The 3 dB bandwidth of the passband was measured as ~0.540 nm in the flat-top band spectrum and ~0.420 nm in the zeroth-order comb spectrum. The 3 dB bandwidth of the flat-top band spectrum is larger by ~28.7%, compared with that of the zeroth-order comb spectrum. In particular, for 20 passband-flattened channels shown in Fig. S2(b), the average passband flatness within one channel and the flatness between channels were measured as ~0.058 and ~0.244 dB, respectively. Moreover, the extinction ratio of the filter was ~20.12 dB or more for a wavelength range of 16 nm.

**
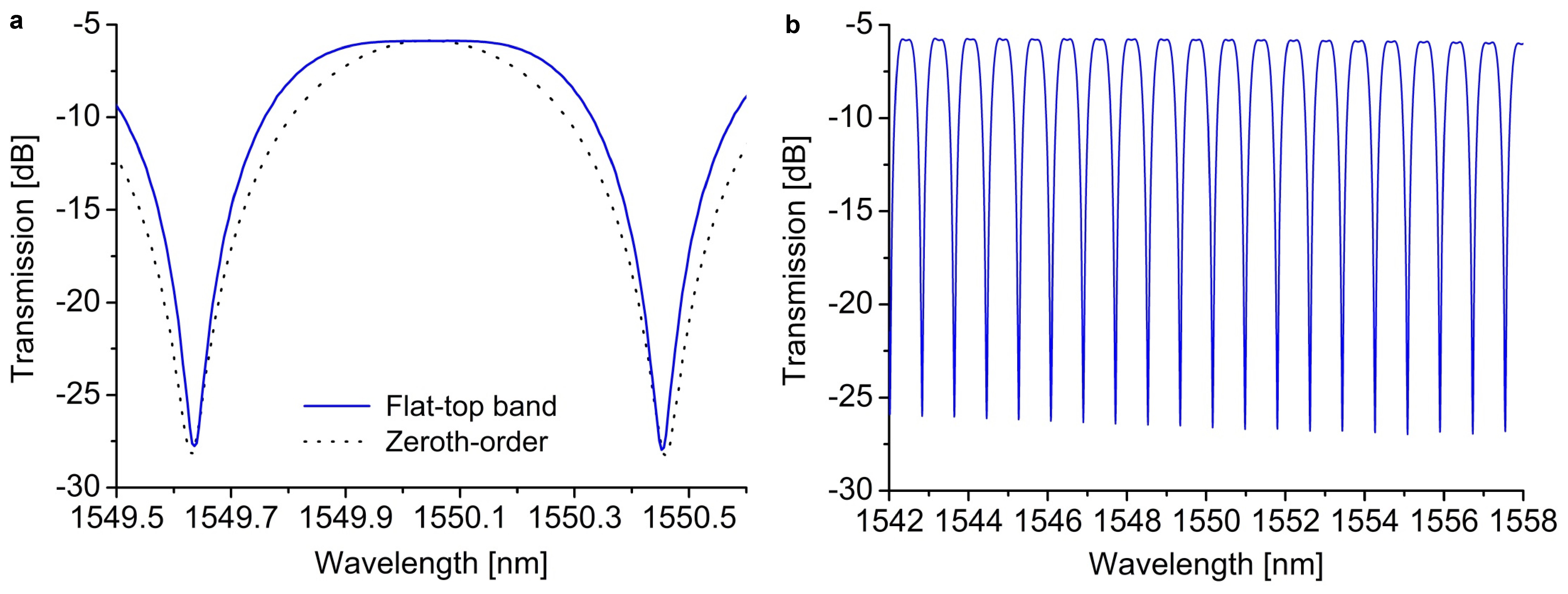
**

**Figure S2.** Transmission spectra measured at (a) narrower (1.1 nm) and (b) wider (16 nm) wavelength ranges. A black dotted curve in (a) indicates the measured zeroth-order comb spectrum for comparison.

**Part 5. Spectral evolution of SOP_in_ and SOP_out_ of each PMF segment at eight selected sets**

Videos S1 and S2 show the spectral evolution of the SOP_in_ and SOP_out_ of PMF 1, respectively, within an FSR Δ*λ_s_* in the CW path for the eight waveplate angle sets (Sets I−VIII) at *θ_p_*_1_ = 0°. Throughout the text, it is assumed that all waveplates used here are achromatic. Thus, the SOP_in_ of PMF 1, determined by the waveplate angle set of HWP 1 and QWP 1, *θ_h_*_1_ and *θ_q_*_1_, is independent of wavelength. While the waveplate angle set changes from Set I to Set VIII in Table 1, the SOP_in_ moves CCW around the *S*_1_ axis on the Poincare sphere along a circle *C*_1_ that has a radius of (1/2)^1/2^ and a center of (2*ε* = 0°, 2*ψ* = 0°) representing LHP. Here, 2*ε* (−90° ≤ 2*ε* ≤ 90°) and 2*ψ* (−180° ≤ 2*ψ* ≤ 180°) are the latitude and longitude of the Poincare sphere, respectively. When the wavelength increases from *λ_A_* to *λ_B_* where *λ_B_* − *λ_A_* = Δ*λ_s_*, the SOP_out_ of PMF 1 makes one CW revolution about the *S*_1_ axis along the circle *C*_1_ on the Poincare sphere. Blue and red open points in Videos S2, S3, and S4 indicate the initial and final points of the spectral revolution of the SOP. The SOP_in_ of PMF 1 exactly becomes the initial point of this spectral evolution at *λ_A_*, denoted by *P*_1_. Hence, *P*_1_ also moves CCW around the *S*_1_ axis on the Poincare sphere along the circle *C*_1_ during the transition of the waveplate angle set from Set I to Set VIII. A set of *θ_h_*_1_ and *θ_q_*_1_ determines the radius and *P*_1_ of this circle, and its center is defined by *θ_p_*_1_. The size and shape of this circular trace affect those of the trajectory formed by the SOP_out_’s of PMF 2.

Videos S3 and S4 show the spectral evolution of the SOP_in_ and SOP_out_ of PMF 2, respectively, within an FSR Δ*λ_s_* in the CW path for the eight waveplate angle sets (Sets I−VIII) at *θ_p_*_1_ = 0°. Over the same wavelength span of Δ*λ_s_* (from *λ_A_* to *λ_B_*), the SOP_in_ of PMF 2 has a circular trace, of which radius is (1/2)^1/2^ and plane is parallel to and distant by (1/2)^1/2^ from a straight line *AB* connecting two points, *A* (2*ε* = 0°, 2*ψ* = 45°) and *B* (2*ε* = 0°, 2*ψ* = −135°) on the Poincare sphere. As the waveplate angle set switches from Set I to Set VIII, this SOP_in_ trace, initially centered at (2*ε* = 0°, 2*ψ* = 135°) at Set I, revolves CCW around the line *AB*, which is realized by HWP 2 and QWP 2. Simultaneously, the SOP_in_ of PMF 2 at *λ_A_*, denoted by *P_in_*, moves CCW on its circular trace according to the revolution, starting from (2*ε* = 0°, 2*ψ* = 180°) at Set I. Here, *P_in_* on each circular trace is determined beforehand by HWP 1 and QWP 1. In short, this SOP_in_ trace spins once CCW around its center while it makes one CCW revolution around the line *AB* with the progress of the waveplate angle set. Any SOP_in_ circular trace of PMF 2 shown in Video S3 makes the SOP_out_ of PMF 2 rotate once CW around the *S*_2_ axis along the trajectory *C*_2_ of a droplet shape with the increase of wavelength from *λ_A_* to *λ_B_*. The same trajectory is obtained for any *θ_p_*_1_ because *θ_p_*_2_ is fixed as 22.5°. The initial point *P*_2_ of the spectral evolution on this trajectory *C*_2_ is directly determined by *P_in_* of the SOP_in_ trace of PMF 2. For example, *P_in_* (2*ε* = 0°, 2*ψ* = 180°) of an SOP_in_ trace centered at (2*ε* = 0°, 2*ψ* = 135°) becomes *P*_2_ on *C*_2_ at Set I, which corresponds to point I in Fig. 5(b). At Set II, *P_in_* of another SOP_in_ trace centered at (2*ε* = 45°, 2*ψ* = 135°) becomes *P*_2_ on *C*_2_, which corresponds to point II in Fig. 5(b). That is, *P*_2_ moves CCW along *C*_2_ around the *S*_2_ axis during the transition from Set I to Set VIII.

Consequently, it is concluded that a phase shift of 0−2*π* can be imposed on the flat-top transmittance function if the following two conditions are satisfied through the control of the four waveplates: (1) The SOP_in_ of PMF 1 should make one CCW rotation around the axis lying in the equatorial plane of the Poincare sphere at a longitude of 2*θ_p_*_1_ along the circular trace whose center and radius are (2*ε* = 0°, 2*ψ* = 2*θ_p_*_1_) and (1/2)^1/2^, respectively. (2) For the SOP_in_ of PMF 2, a circular trace of a radius (1/2)^1/2^, which results from spectral evolution over a wavelength range from *λ_A_* to *λ_B_* (Δ*λ_s_* = *λ_B_* − *λ_A_*), should make one CCW revolution around the axis lying in the equatorial plane at 2*ψ* = 45°, i.e., the line *AB*, at the same rate as the above rotation rate, while maintaining a distance of (1/2)^1/2^ parallel to the line *AB*. Simultaneously, this revolution should start from the circle centered on (2*ε* = 0°, 2*ψ* = 135°) when the initial point of the above SOP_in_ trace of PMF 1 is (2*ε* = 45°, 2*ψ* = 2*θ_p_*_1_), with the initial point of the spectral evolution, or *P_in_*, on each circle staying on *C*_2_ during the revolution.
